# Posterior fossa epidural hematoma: A 6-year management experience

**DOI:** 10.12669/pjms.39.1.6408

**Published:** 2023

**Authors:** Seema Sharafat, Farooq Azam, Zahid Khan

**Affiliations:** 1 Seema Sharafat, FCPS., Department of Neurosurgery, Medical and Teaching Institute, Lady Reading Hospital, Peshawar, Pakistan; 2 Farooq Azam, FCPS, Department of Neurosurgery, Medical and Teaching Institute, Lady Reading Hospital, Peshawar, Pakistan; 3 Zahid Khan, FCPS, Department of Neurosurgery, Medical and Teaching Institute, Lady Reading Hospital, Peshawar, Pakistan

**Keywords:** Posterior Fossa, Epidural, Hematoma, Glasgow Coma Scale (GCS), CT-Scan

## Abstract

**Objective:**

Through this study, we sought to evaluate the management of posterior fossa extradural hematoma (PFEDH).

**Methods:**

An observational study was conducted at the Neurosurgery Department of Lady Reading Hospital in Peshawar from January 2015 to December 2020. All patients who had a traumatic acute extradural hematoma (EDH) of the posterior fossa were included, irrespective of age and gender. The clinical predictors and outcomes were assessed, including the CT-scan findings and Glasgow Coma Scale (GCS) score.

**Results:**

A total of 104 cases with posterior fossa extradural hematoma were identified from 1252 extradural hematoma patients admitted during the study period. The mean age of the enrolled patients was 18.17 ± 14.31 years. Most of the patients were male (65.39%) and belonged to the pediatric age group, i.e., < 15 years (60.6%). CT scan brain was done in all the cases for diagnosis. In 68.3% of cases, an associated occipital bone fracture was observed. Surgery was done in almost 71.2% of cases, and most of the patients experienced good recovery after surgery, as indicated by the GOS score. Linear regression model revealed that treatment (β=-0.20, p=0.038), time duration between surgery and trauma (β=0.43, p=0.000) and GCS category (β=-0.47, p=0.000) were significantly associated with PFEDH outcomes.

**Conclusion:**

In conclusion, PFEDH was frequent among males and the pediatric age group. Serial CT brain is highly recommended in all suspected cases for early diagnosis.

## INTRODUCTION

Posterior fossa extradural hematoma (PFEDH) is a rare neurosurgical entity, resulting in rapid clinical deterioration. The accumulation of blood in a potentially small space between the dura and occipital bone cause compression of the brain stem.^1^,^2^ These hematomas constitute almost 4% to 12.9% of all extradural hematoma cases.^3^

The extradural hematomas of the posterior fossa may be categorized as acute or delayed defined with respect to the brainstem compression appearing within or after 24 hours of injury, respectively.^4^,^5^ Acute PFEDH is characterized by medullar failure, occipital trauma associated with severe pain in the nuchal area, altered consciousness followed by rapid brainstem compression, respiratory depression, and subsequent death if not treated timely and appropriately.^6^ Whereas, headache, neck pain, dysfunction of lower cranial nerve in response to PF lesions and cerebellar signs.^5^ The simultaneous systemic traumatic lesions cause a subsequent increase in intracranial pressure (ICP) and traumatic intracranial lesions that is primarily responsible for the development of delayed PFEDH.^7^

The clinical features of PFEDH are nonspecific; it may include occipital headache, vomiting due to raised intracranial pressure, and decreased consciousness level leading to death. The skull fractures must be considered as a predisposing factor for the development of delayed extradural hematomas.^8^ A few studies also define delayed extradural hematoma development in relation to bleeding of venous origin.^9^ Unilateral mydriasis is among the most frequent misleading signs observed among PDEFH patients, which could be explained by the deformation of tentorial hiatus and oculomotor nerve compression causing cerebellum upward herniation.^6^

Computed tomography (CT) of the brain has been recognized as the vital diagnostic modality for PFEDH. Furthermore, the condition can be treated either surgically or conservatively depending upon the clinical and radiological parameters.^10^,^11^ Coleman and Thompson reported the first successful surgery for PFEDH in 1941.^12^

Unlike temporal extradural hematoma, the accumulation of blood in the posterior fossa is relatively slow, and the development of EDH is delayed. This is because the origin of bleeding is venous and not arterial. The primary management complication seen in PFEDH is that even a small hematoma requires surgical evacuation.^2^ Furthermore, the condition is frequently misdiagnosed, i.e., an insignificant supratentorial hematoma is usually evacuated overlooking the life-threatening PFEDH.^13^ This study will add valuable information about PFEDH, as there is not much local literature highlighting the clinical parameters and outcomes of the disease.

## METHODS

This observational study was conducted at the Neurosurgery Department, Lady Reading Hospital, Peshawar, from January 2015 to December 2020. The ethical approval was obtained from the institutional ethics committee (Reference no. 37/LRH/MTI; Dated 09-02-2021). During the six years’ period, a total of 1252 patients with extradural hematoma were admitted to the study site, and out of them, PFEDH was found in 104 patients whose data was included in the final analysis. All PFEDH patients were included in the study irrespective of age and gender; more than 50% of the enrolled cases were < 15 years of age and were identified as pediatric cases of PFEDH. Patients who had hematoma due to bleeding disorders or had post-operative hematoma were excluded from the study sample.

The patient’s clinical data, treatment type, and outcomes of cranial CT scan on admission were obtained from the hospital records and studied retrospectively. The decision regarding the therapeutic intervention to be used primarily depended on the clinical condition of the patients and CT scan findings. The patients with fourth ventricular compression or displacement or obstructive ventriculomegaly and those having a clot volume of ≥ 15 mL were recommended for surgical intervention. While those with no associated intracranial or midline shift lesions or those having hematomas with relatively smaller volume < 10 mL were treated conservatively. All the patients were followed up for one month after surgery.

**Table-I:** Baseline characteristics of patients with posterior fossa extradural hematoma.

Variables		n=104
Age (years)		18.17±14.31
	< 15 years	63(60.6)
	> 15 years	41(39.4)
Gender	Male	68(65.39)
	Female	36(34.61)

Moreover, the level of consciousness was assessed using the Glasgow coma scale (GCS). After surgery, the patient’s outcome was assessed using the Glasgow outcome scale (GOS). GOS score four and five were considered good recovery outcomes, GOS 2 and 3 indicated poor outcomes, and OS one indicated mortality.

The statistical analysis was performed on SPSS version 22.0. Descriptive statistics was used, all continuous variables were presented as mean and standard deviation, while frequencies and percentages were used to display categorical variables. Chi-square test and linear regression analysis was performed to determine the factors associated with PFEDH outcomes, where p<0.05 was considered statistically significant.

## RESULTS

Out of 1252 extradural hematoma patients admitted to the Department of Neurosurgery, Lady Reading Hospital, Peshawar, there were 104 cases of PFEDH. The mean age of the study participants was 18.17 ± 14.31 years.

CT brain was done in all the cases to diagnose PFEDH. In 71.2% of all enrolled cases, the hematoma was picked by the CT scan performed on admission. While in 28.8% of cases, the repeated CT scan taken after 6-8 hours of arrival revealed hematoma. The occipital bone fracture was found in 68.3% of cases. Most of the patients underwent surgery (posterior fossa craniotomy or craniotomy) to evacuate hematoma (71.2%). Five patients were observed having a post-operative wound infection, and two patients also required re-evacuation of hematoma after two days.

**Table-II:** Pre-op GCS score and post-op outcomes of posterior fossa epidural hematoma.

Variables		N (%)
GCS at admission	13-15 (Mild)	48(46.2)
	9-12 (Moderate)	23(22.1)
	3-8 (Severe)	33(31.7)
GOS after surgery	4 & 5 (Good)	86(82.69)
	2 & 3 (Poor)	09(6.65)
	1 (Mortality)	09(8.65)

*GCS-Glasgow Coma Scale; GOS-Glasgow Outcome Scale.

**Table-III:** Factors affecting the outcomes of PFEDH.

Variables		GOS [N(%)]			Standardized coefficients (β)	P-value

		Good	Poor	Mortality		
Gender	Female	32(37.2)	1(11.1)	3(33.3)	-0.07	0.429
	Male	54(62.8)	8(88.9)	6(66.7)		
Time duration between surgery & trauma	< 12 hours	32(37.2)	-	-	0.43	0.000*
	12-24 hours	38(44.2)	3(33.3)	2(22.2)		
	> 24 hours	16(18.6)	6(66.7)	7(77.8)		
Associated fracture	Yes	61(70.9)	6(66.7)	4(44.4)	0.15	0.125
	No	25(29.1)	3(33.3)	5(55.6)		
Treatment	Surgery	57(66.3)	9(100.0)	8(88.9)	-0.20	0.038*
	Conservative	29(33.7)	-	1(11.1)		
GCS category	Severe	17(19.8)	8(88.9)	8(88.9)	-0.47	0.000*
	Moderate	22(25.6)	1(11.1)	-		
	Mild	47(54.7)	-	1(11.1)		

* p<0.05 is considered statistically significant.

A total of 48 patients (46.2%) were admitted at the study site with the GCS scores of 13-15 (mild), 23 patients (22.1%) had a GCS score between 9-12 (moderate), and 33 patients (31.7%) had 6-8 (severe) GCS score at the time of admission. Of the total, 9 (8.65%) patients died (GOS 1), and 86 (82.69%) patients displayed good recovery (GOS 4 & 5).

Time duration between surgery and trauma, treatment and GCS category were significantly associated with PFEDH outcomes (p<0.05).

## DISCUSSION

PFEDH is rare but significant neuropathology, as delayed diagnosis and management lead to substantial mortality and morbidity.^14^ Studies have reported that PFEDH is more common in the male gender and the pediatric age group.^1^,^15^ Up to 23.3% of children with trauma developed PFEDH, while the incidence is comparatively less, i.e., only 12.7% of adults are known to suffer from the disease as per the Suyama series.^16^ Our results were also consistent with these observations, more than 60% of the managed patients at the study site were males, and almost 60.6% were ≤ 15 years of age. This increased susceptibility among males might be because they are more prone to trauma as they have an increased number of outdoor chores than females. While among children, the increased vulnerability to injury and the higher incidence is mainly due to the anatomical proximity in the dura and venous sinus.^9^,^10^

Cranial CT scan contributes to the early diagnosis of PFEDH; it is an effective imaging technique and has a short acquisition time. It is evident from the existing literature that the CT efficiently determines the size of the hematoma and associated occipital bone fracture; it also tells us about compression of the posterior fossa structures and hydrocephalous, which may need surgical intervention.^17^ Therefore, sustaining the management standards for effective and timely diagnosis, cranial CT scan findings were obtained for all the enrolled cases.

The radiologic investigations suggest that occipital fractures are usually present in around 85% of PFEDH cases.^13^ The reported incidence of occipital skull fractures associated with posterior fossa extradural hematoma ranges between 78-93%.^18^ A study reported 87.5% cases PFEDH linked occipital skull fractures.^17^ Another study reported an incidence rate of 72%.^19^ We found a relatively lesser frequency of occipital bone fracture, i.e., 68.3 %, associated with posterior fossa hematoma.

The PFEDH treatment may be surgical or conservative. Although surgical evacuation is the gold standard of traditional treatment, it is practiced only if the hematoma is large and causes mass effect.^2^,^20^ In our study, almost 71.2% of the patients were treated surgically, and the outcome was good in 82.69% of the cases. A similar study reported a slightly higher recovery rate (89.8%) than that reported in the present study.^15^ Roka et al. studied 43 patients with PFEDH, operated on 33 of them, and reported good recovery in 81.8%.^21^ While a few also reported decreased recovery. Malik et al. studied 61 cases, and only 59% of their patients had a good recovery.^13^

Previously, PFEDH was reported with high mortality; some old studies reported mortality up to 50% of the cases.^22^ However, in the post-CT scan era, the mortality has evidently decreased with better outcomes due to early diagnosis and treatment.^2^,^23^ Jang et al. studied 34 patients and reported only 5.3% mortality.^24^ In contrast, we have observed a high mortality rate, i.e., 8.65%. The reason for this may be delayed admission, severe head injuries, and low GCS in the enrolled patients.

Therefore, it is established that PFEDH can result in good outcomes with appropriate and timely diagnosis and management.^1^,^25^ Thus, admission cranial CT scan and GCS are the most important clinical modalities defining the immediate diagnosis and outcomes among PFEDH patients.

### Limitations:

Among the major limitation of the study was its retrospective nature. The only available data in the medical records were used for the study purpose. Hence, there is a lack of data regarding the potential confounding factors.

### Recommendations:

Large-scale, multicenter prospective studies are recommended to investigate this clinical concern. All suspected cases must undergo early and repeated CT scans. We advise against delaying surgical excision of a hematoma once it has been determined that it needs to be removed in order to lower morbidity and death.

## CONCLUSION

PFEDH frequently affects the male gender and children. For good outcomes, early diagnosis and evacuation are essential. Serial CT brain should be done in all suspected cases for the timely diagnosis to limit the disease-associated mortality. Conservative treatment is a viable option among patients with low EDH volumes, but surgical evacuation is highly recommended for critical cases with high volumes.
